# Supplementary material for: Single-cell spatial proteomic imaging for human neuropathology
Source: Acta Neuropathol Commun. 2022 Nov 4;10:158. doi: 10.1186/s40478-022-01465-x (PMC9636771; doi:10.1186/s40478-022-01465-x)
Supplement: Supplementary file 1 — Additional file 1: Supplementary Figures and legends S1 through S6. [file 40478_2022_1465_MOESM1_ESM.pdf]

SUPPLEMENTAL FIGURES

This includes:

Figs. S1 to S6

Figure S1

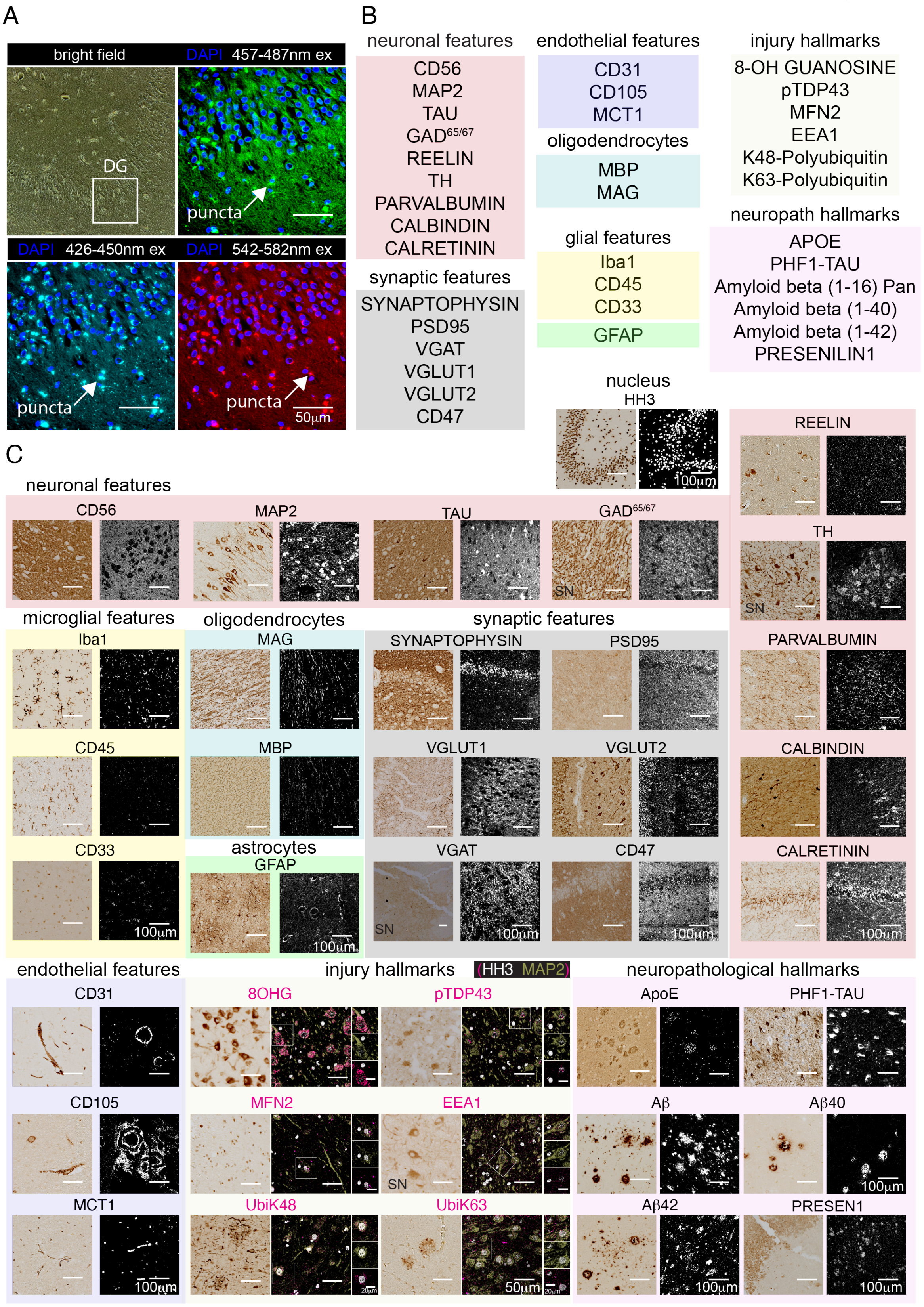

A
bright field
DAPI 457-487nm ex
DG
puncta
DAPI 426-450nm ex
DAPI 542-582nm ex
puncta
puncta
50µm
B
neuronal features
CD56
MAP2
TAU
GAD65/67
REELIN
TH
PARVALBUMIN
CALBINDIN
CALRETININ
synaptic features
SYNAPTOPHYSIN
PSD95
VGAT
VGLUT1
VGLUT2
CD47
endothelial features
CD31
CD105
MCT1
oligodendrocytes
MBP
MAG
glial features
Iba1
CD45
CD33
GFAP
injury hallmarks
8-OH GUANOSINE
pTDP43
MFN2
EEA1
K48-Polyubiquitin
K63-Polyubiquitin
neuropath hallmarks
APOE
PHF1-TAU
Amyloid beta (1-16) Pan
Amyloid beta (1-40)
Amyloid beta (1-42)
PRESENILIN1
nucleus
HH3
100µm
C
neuronal features
CD56
MAP2
TAU
GAD65/67
SN
REELIN
TH
SN
microglial features
Iba1
CD45
CD33
100µm
oligodendrocytes
MAG
MBP
astrocytes
GFAP
100µm
synaptic features
SYNAPTOPHYSIN
PSD95
VGLUT1
VGLUT2
VGAT
CD47
SN
100µm
100µm
PARVALBUMIN
CALBINDIN
CALRETININ
100µm
endothelial features
CD31
CD105
MCT1
100µm
injury hallmarks
(HH3 MAP2)
8OHG
pTDP43
MFN2
EEA1
SN
UbiK48
UbiK63
50µm
neuropathological hallmarks
ApoE
PHF1-TAU
Aβ
Aβ40
100µm
Aβ42
PRESEN1
100µm
100µm

Figure S1. Validation of MIBI-TOF antibody panel on FFPE archival brain tissue.
(A) Autofluorescence photomicrographs of an unstained human hippocampus section under bright field, green (457-487nm excitation), cyan (426-450nm excitation) and red (542-582nm excitation) fluorescence channels. (B) List of brain specific targets used for MIBI-TOF analysis. (C) Immunohistochemical and MIBI-TOF antibody validation and cross comparison of chromogenic and spectral images.

Figure S2

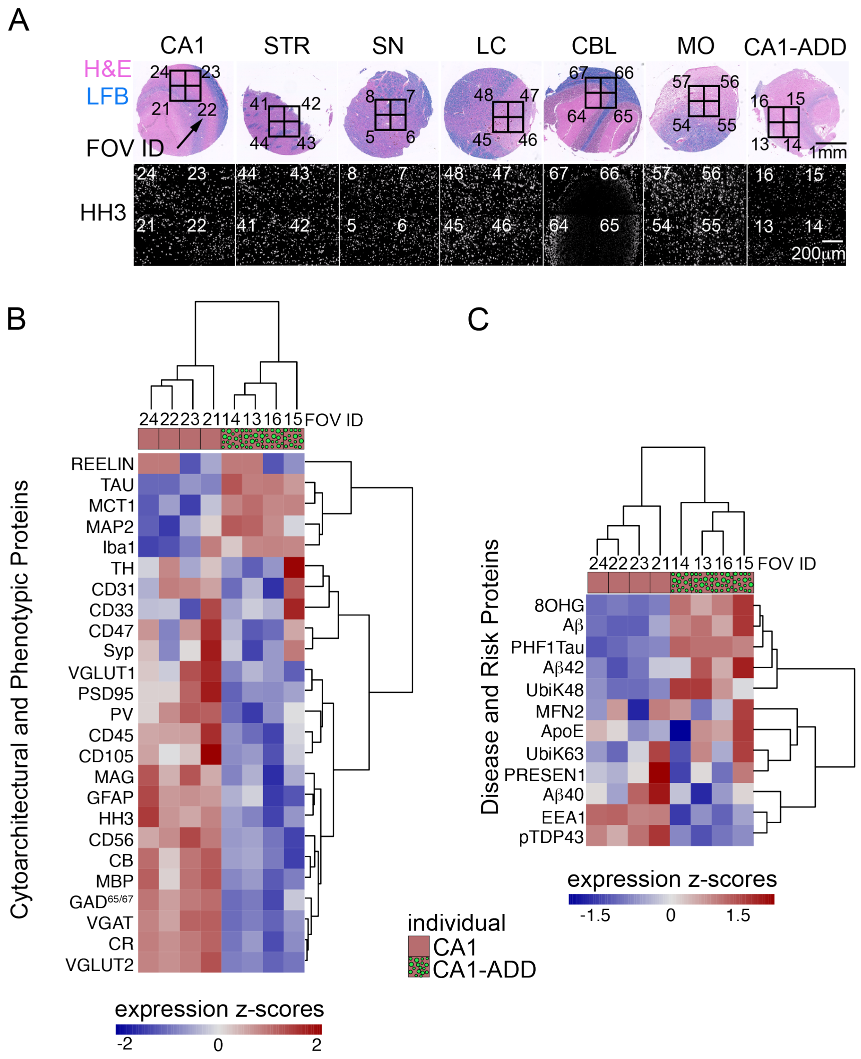
A
CA1
STR
SN
LC
CBL
MO
CA1-ADD
H&E
LFB
FOV ID
HH3
1mm
200µm
B
C
FOV ID
Cytoarchitectural and Phenotypic Proteins
REELIN
TAU
MCT1
MAP2
Iba1
TH
CD31
CD33
CD47
Syp
VGLUT1
PSD95
PV
CD45
CD105
MAG
GFAP
HH3
CD56
CB
MBP
GAD65/67
VGAT
CR
VGLUT2
expression z-scores
-2
0
2
individual
CA1
CA1-ADD
Disease and Risk Proteins
8OHG
Aβ
PHF1Tau
Aβ42
UbiK48
MFN2
ApoE
UbiK63
PRESEN1
Aβ40
EEA1
pTDP43
expression z-scores
-1.5
0
1.5

Figure S2. Global Expression Analysis of Multiplex Proteomic Data Stratifies cognitivenormal and ADD CA1 cores.
(**A**) Photomicrograph of brain TMA cores stained standard histological Luxol Fast Blue (LFB) for myelin with hematoxylin and eosin (H&E) for cells (top). Green boxes depict the rastered area (4x4, 500μm^2) and each FOV is assigned an ID. The spectral images of HH3 show the presence and distribution of nuclei in each FOV and number represent the IDs for each FOV (bottom). (**B-C**) of mean z-score distribution of pixel expression of proteins per rastered FOV (row normalized). Columns and rows are hierarchically clustered (Euclidean distance). Variance between and among FOVs are stratified in an unsupervised manner into normative and disease. Columns and rows are hierarchically clustered (Euclidean distance). (**B**) Showing cytoarchitectural changes between normative (CA1) and disease (CA1-ADD) in the CA1 TMA cores. (**C**) Showing AD disease and risk target protein changes between normative and disease in the CA1 TMA cores. Abbreviations: CA1, Cornu Ammonis 1; STR, Striatum; SN, Substantia Nigra; LC, Locus Coeruleus; CBL, Cerebellum; MO, Medulla Oblongata; CA1-ADD, Cornu Ammonis 1-AD dementia.

Figure S3

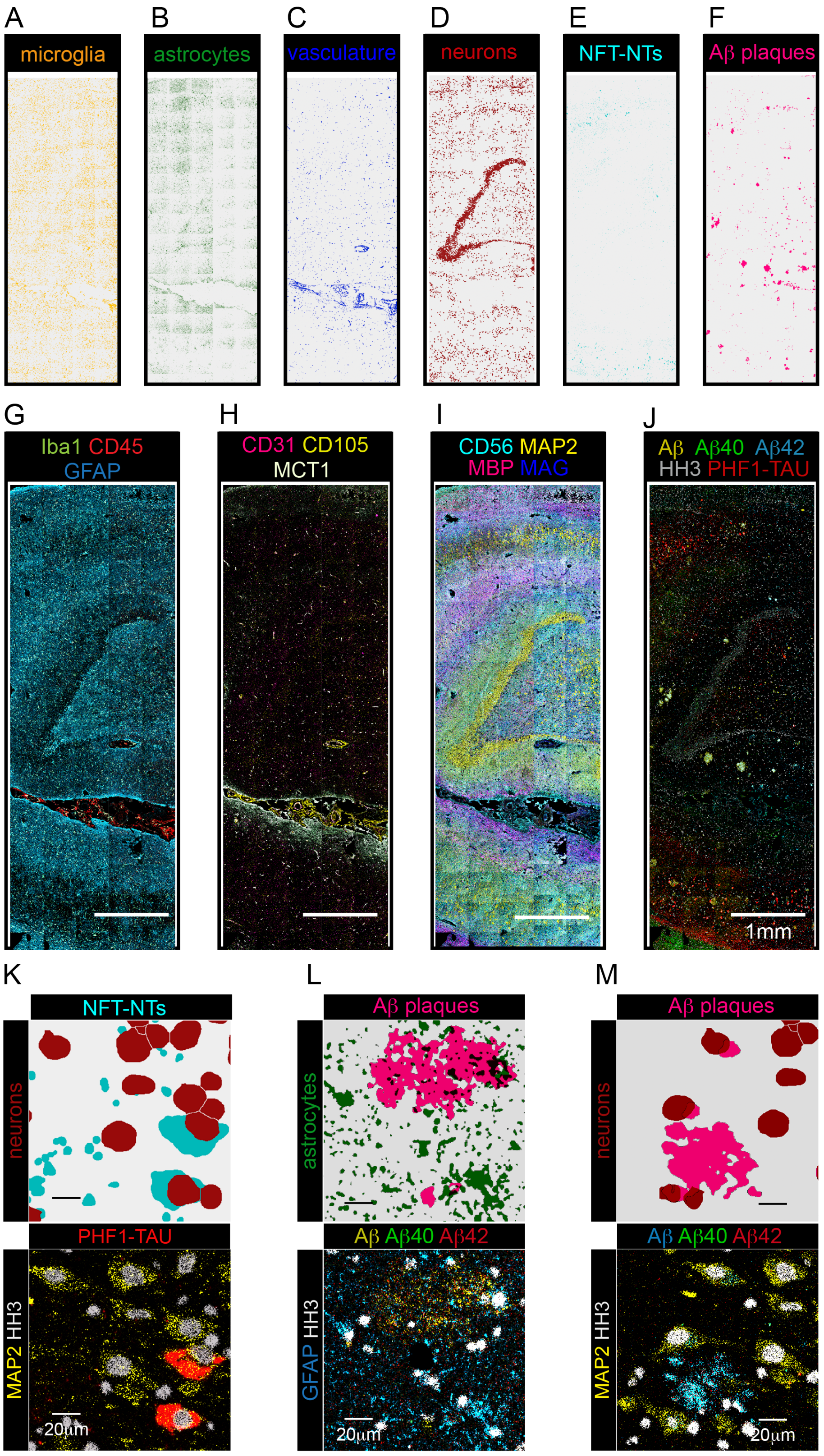
A
microglia
B
astrocytes
C
vasculature
D
neurons
E
NFT-NTs
F
Aβ plaques
G
Iba1 CD45
GFAP
H
CD31 CD105
MCT1
I
CD56 MAP2
MBP MAG
J
Aβ Aβ40 Aβ42
HH3 PHF1-TAU
1mm
K
NFT-NTs
neurons
PHF1-TAU
MAP2 HH3
20µm
L
Aβ plaques
astrocytes
Aβ Aβ40 Aβ42
GFAP HH3
20µm
M
Aβ plaques
neurons
Aβ Aβ40 Aβ42
MAP2 HH3
20µm

Figure S3. Cell Phenotype Maps (CPM) of Segmented features in ADD hippocampus. **(A-F)** Individual Cell Phenotype Maps (CPM) of nuclear and object segmented masks labelled by their phenotypes. **(G-J)** MIBI-TOF pseudo-colored spectral images for the different cells which are identified by their pan-markers (GFAP: astrocytes, Iba1-CD45: microglia, CD31-CD105-MCT1: vessels, MAP2: neurons, CD45: immune cells, MBP and MAG: Oligodendrocytes). **(K-M)** Segmented mask overlaysof cell and proteopathy phenotypes with corresponding MIBI-TOF pseudo-colored spectral images.

Figure S4A-C

cognitively normal no dementia (CN)

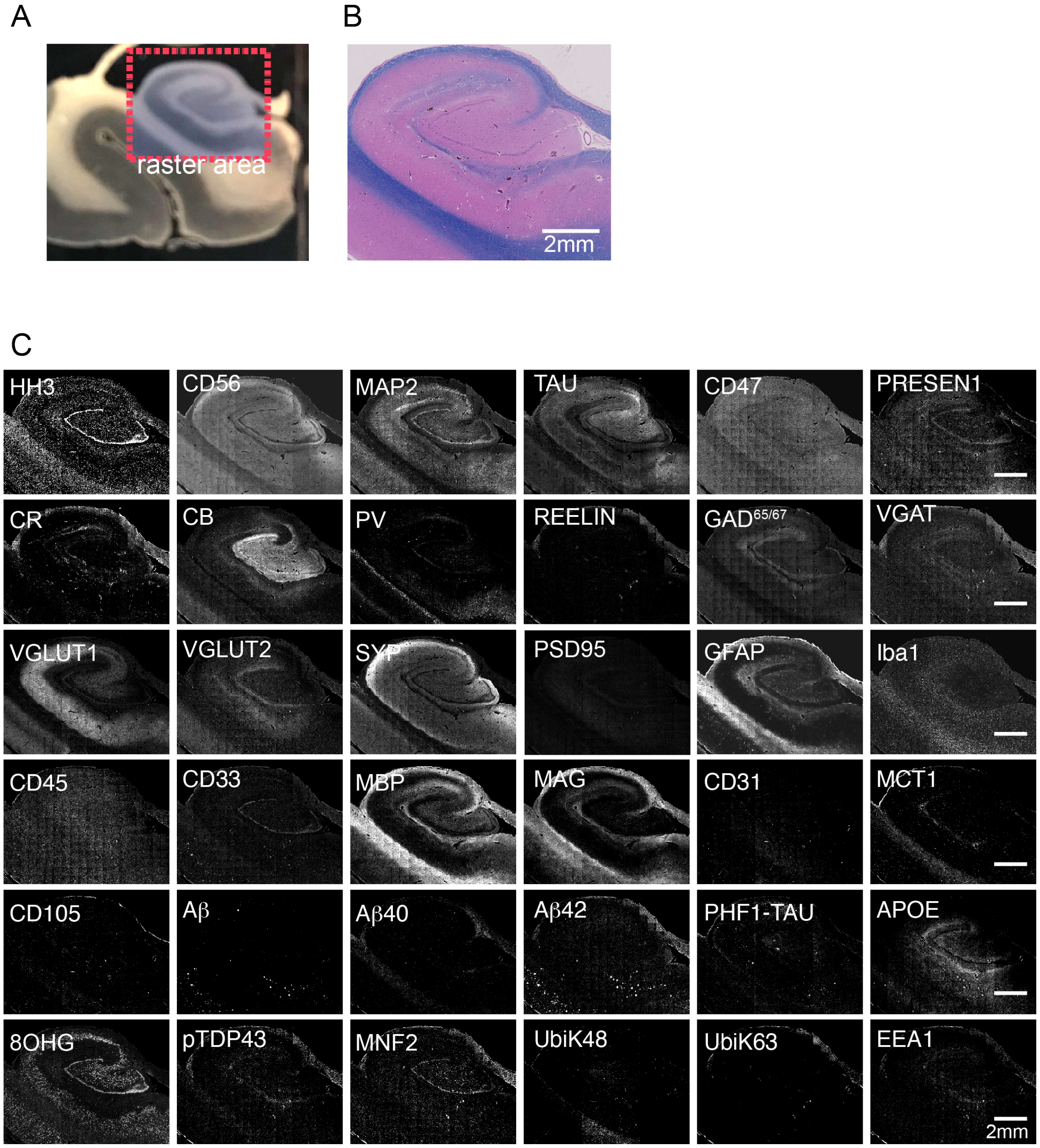

cognitively impaired no dementia (CIND)

D

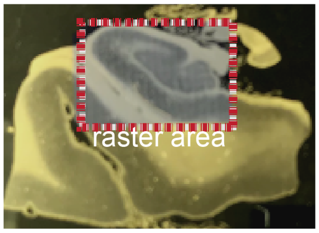

E

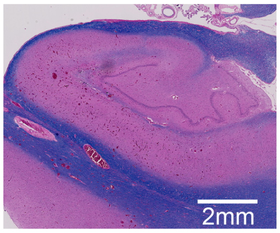

F

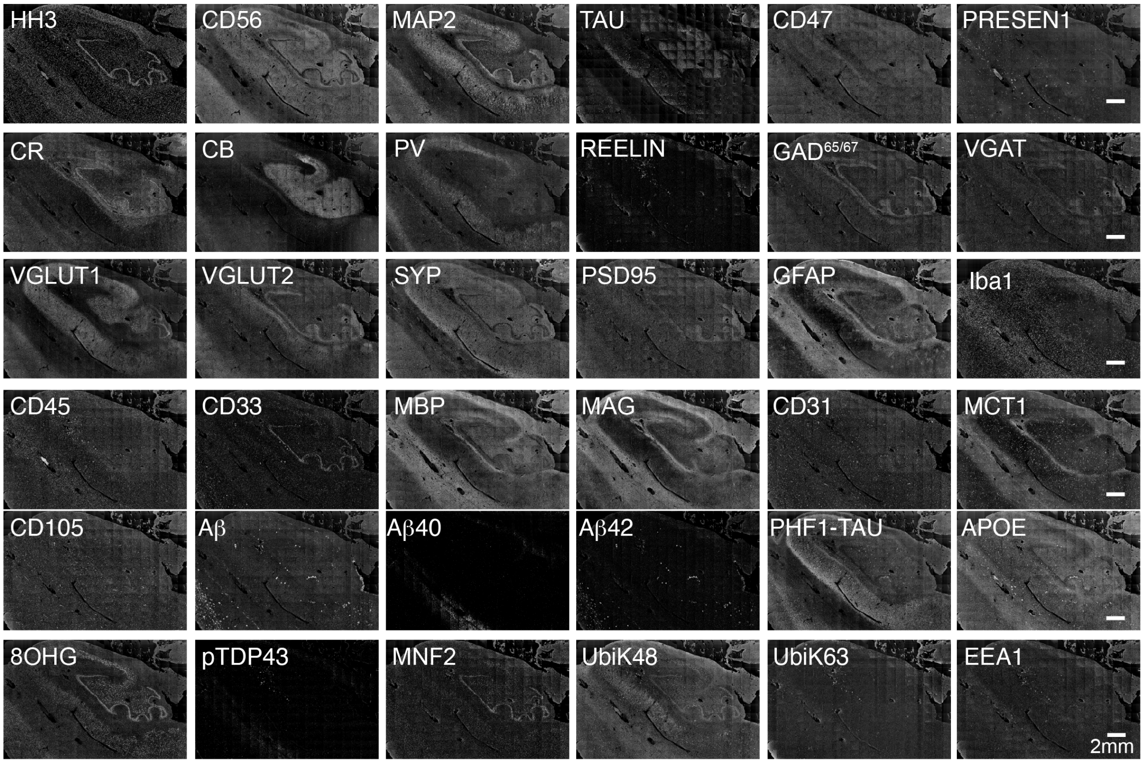

Figure S4G-I

cognitively impaired with dementia (ADD)

G

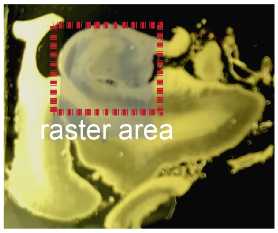

H

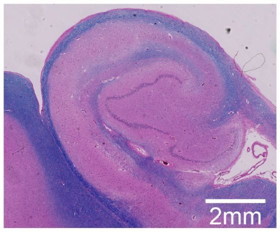

I

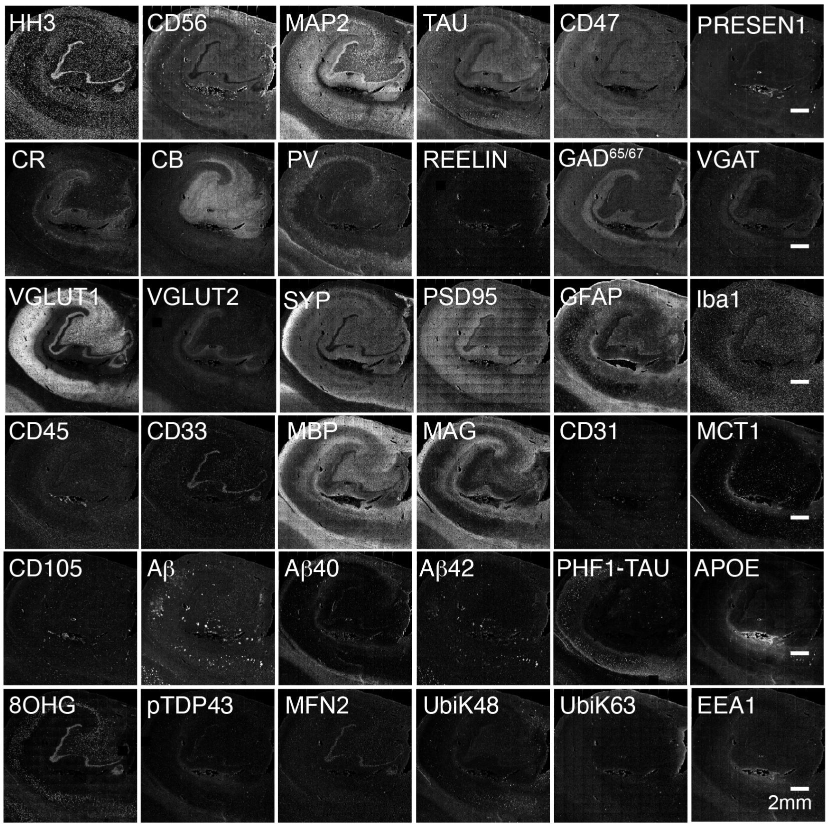

J

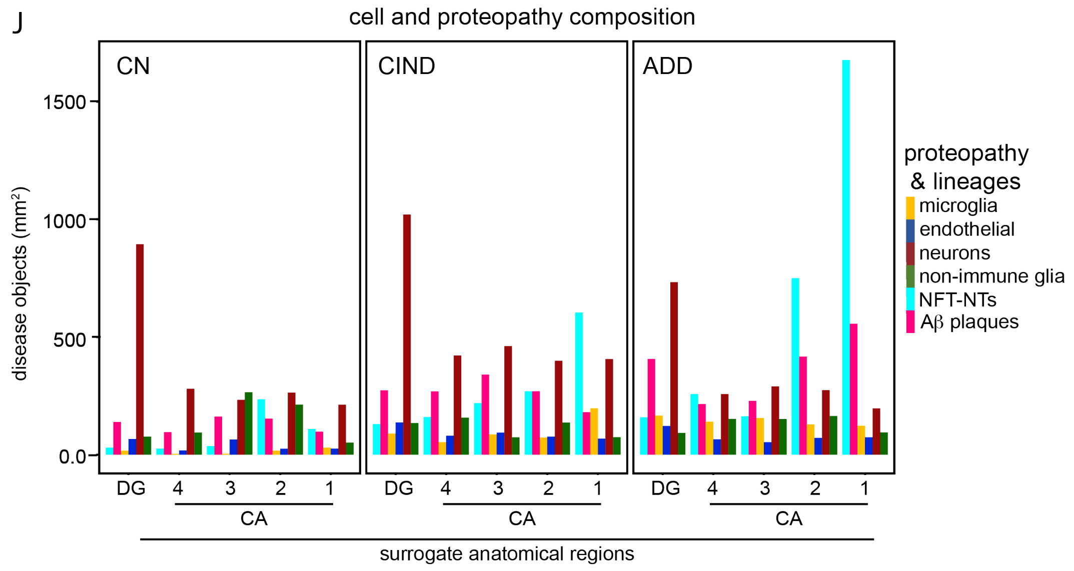
cell and proteopathy composition
CN
CIND
ADD
1500
1000
500
0.0
disease objects (mm2)
DG
4
3
2
1
CA
surrogate anatomical regions
proteopathy & lineages
microglia
endothelial
neurons
non-immune glia
NFT-NTs
Aβ plaques

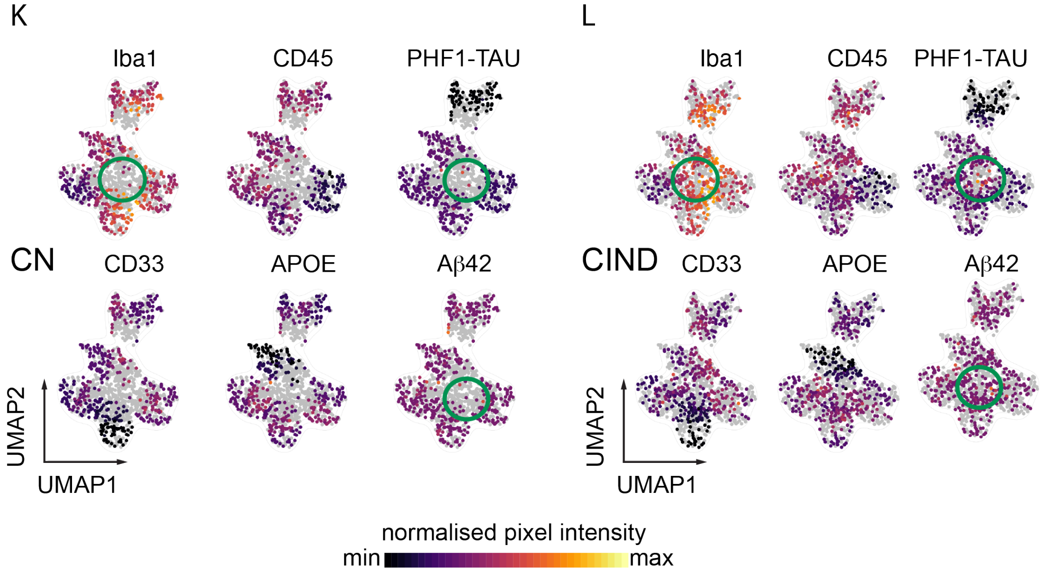
K
Iba1
CD45
PHF1-TAU
CN
CD33
APOE
Aβ42
UMAP2
UMAP1
L
Iba1
CD45
PHF1-TAU
CIND
CD33
APOE
Aβ42
UMAP2
UMAP1
normalised pixel intensity
min
max

Figure S4. Single-plex spectral images of CN, CIND and ADD and their breakdown of cell and proteopathy characteristics in anatomical regions. **(A, D, G)** Archival mid-section hippocampi from CN, CIND, or ADD on conductive gold-slides. Red dashed boxes show rastered regions respectively. **(B, E, H)** Serial sections stained with standard histological Luxol Fast Blue (LFB) for myelin with hematoxylin and eosin (H&E) for cells. **(C, F, I)** Single plex MIBI spectral images for all 36 targets that were simultaneously acquired. Images for CN is of 300 FOVs tiled; CIND is of 260 FOVs tiled, ADD is of 196 tiled FOVs. Each FOV is 500µm^2. Images are gray scaled. **(J)** Normalized counts of cells and proteopathies within each mm^2 of tissue across each hippocampal anatomical region and individual. **(K)** UMAP projections of CA1 microglia phenotyping channels in CN. Gray representsCA1 microglia from ADD, CIND samples. **(L)** UMAP projections of CA1 microglia phenotyping channels in CIND. Gray represents CA1 microglia from ADD, CN samples. Projection calculatedusing phenotypic markers. CIND and ADD are subsampled so that all conditions are represented by 447 microglia, the total number of CA1 microglia in the CN condition.

Figure S5 ↑

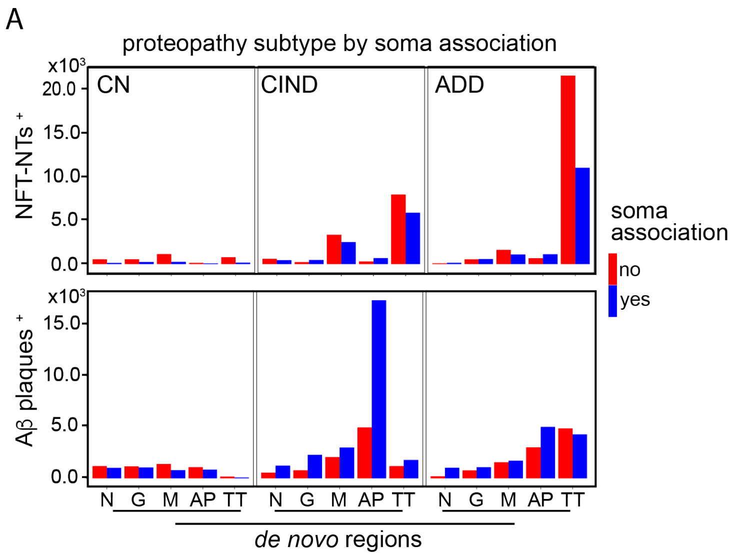

A
proteopathy subtype by soma association
CN
CIND
ADD
x10³
20.0
15.0
10.0
5.0
0.0
NFT-NTs⁺
x10³
15.0
10.0
5.0
0.0
Aβ plaques⁺
soma association
no
yes
N G M AP TT
N G M AP TT
N G M AP TT
de novo regions

Figure S5. Breakdown of cell and proteopathy characteristics in de novo regions.
(A) Raw counts of proteopathies associated with a cell soma or not across the de novo regions.Highest counts of Aβ plaques found in AP region within CIND (majority soma associated), highest counts of tau NFT-NTs found within TT in ADD (majority not soma associated). **(B)** Normalized meanexpression of non-immune glia, microglia, endothelial cells, and neurons with associated phenotyping channels. Broken down across the *de novo* regions.

Figure S6A-C ↑

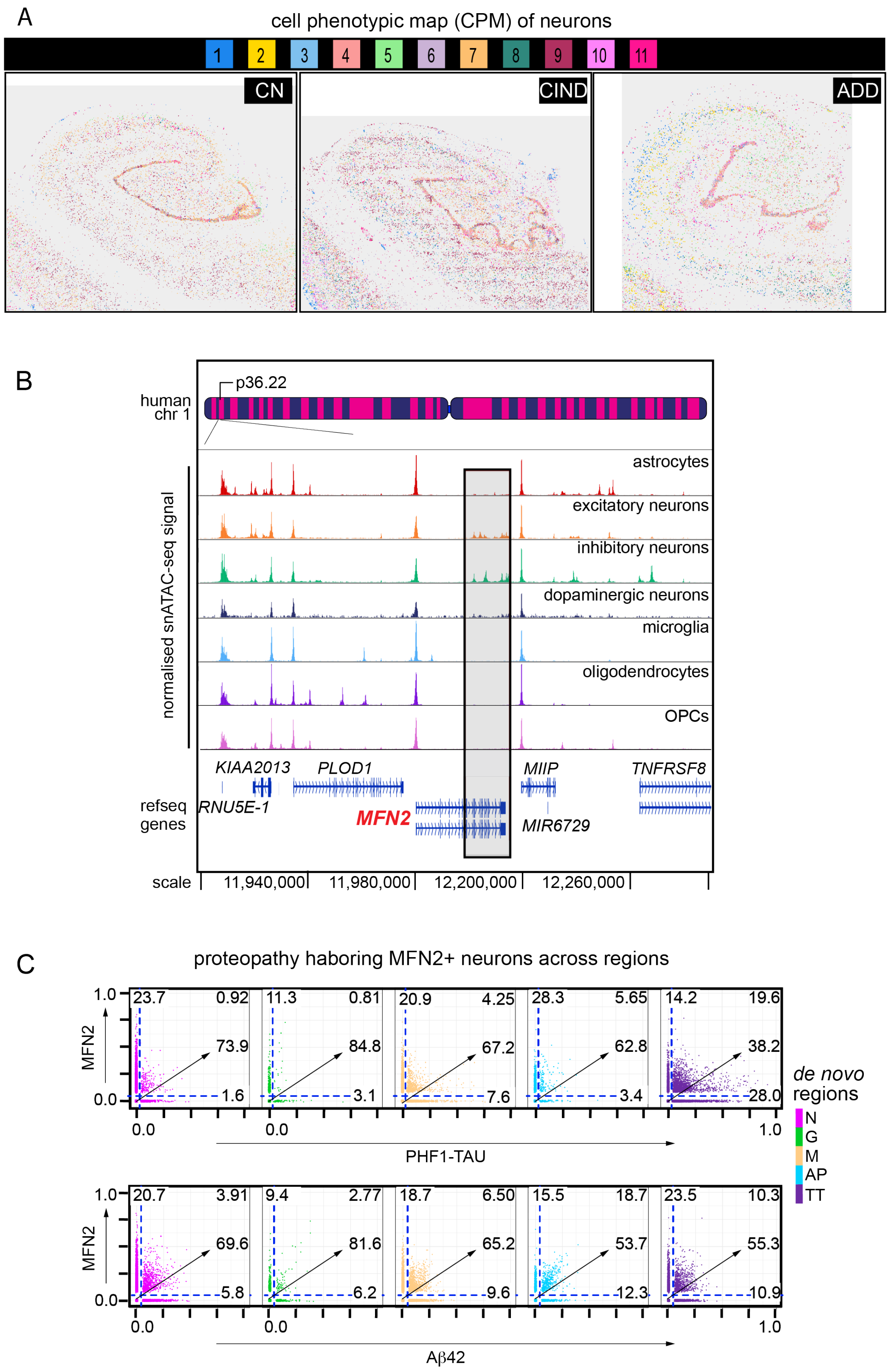
A
cell phenotypic map (CPM) of neurons
1 2 3 4 5 6 7 8 9 10 11
CN
CIND
ADD
B
human chr 1
p36.22
normalised snATAC-seq signal
astrocytes
excitatory neurons
inhibitory neurons
dopaminergic neurons
microglia
oligodendrocytes
OPCs
refseq genes
KIAA2013
PLOD1
RNU5E-1
MFN2
MIIP
MIR6729
TNFRSF8
scale
11,940,000
11,980,000
12,200,000
12,260,000
C
proteopathy haboring MFN2+ neurons across regions
MFN2
PHF1-TAU
23.7 0.92 73.9 1.6
11.3 0.81 84.8 3.1
20.9 4.25 67.2 7.6
28.3 5.65 62.8 3.4
14.2 19.6 38.2 28.0
Aβ42
20.7 3.91 69.6 5.8
9.4 2.77 81.6 6.2
18.7 6.50 65.2 9.6
15.5 18.7 53.7 12.3
23.5 10.3 55.3 10.9
1.0
0.0
de novo regions
N
G
M
AP
TT

Figure S6D-F

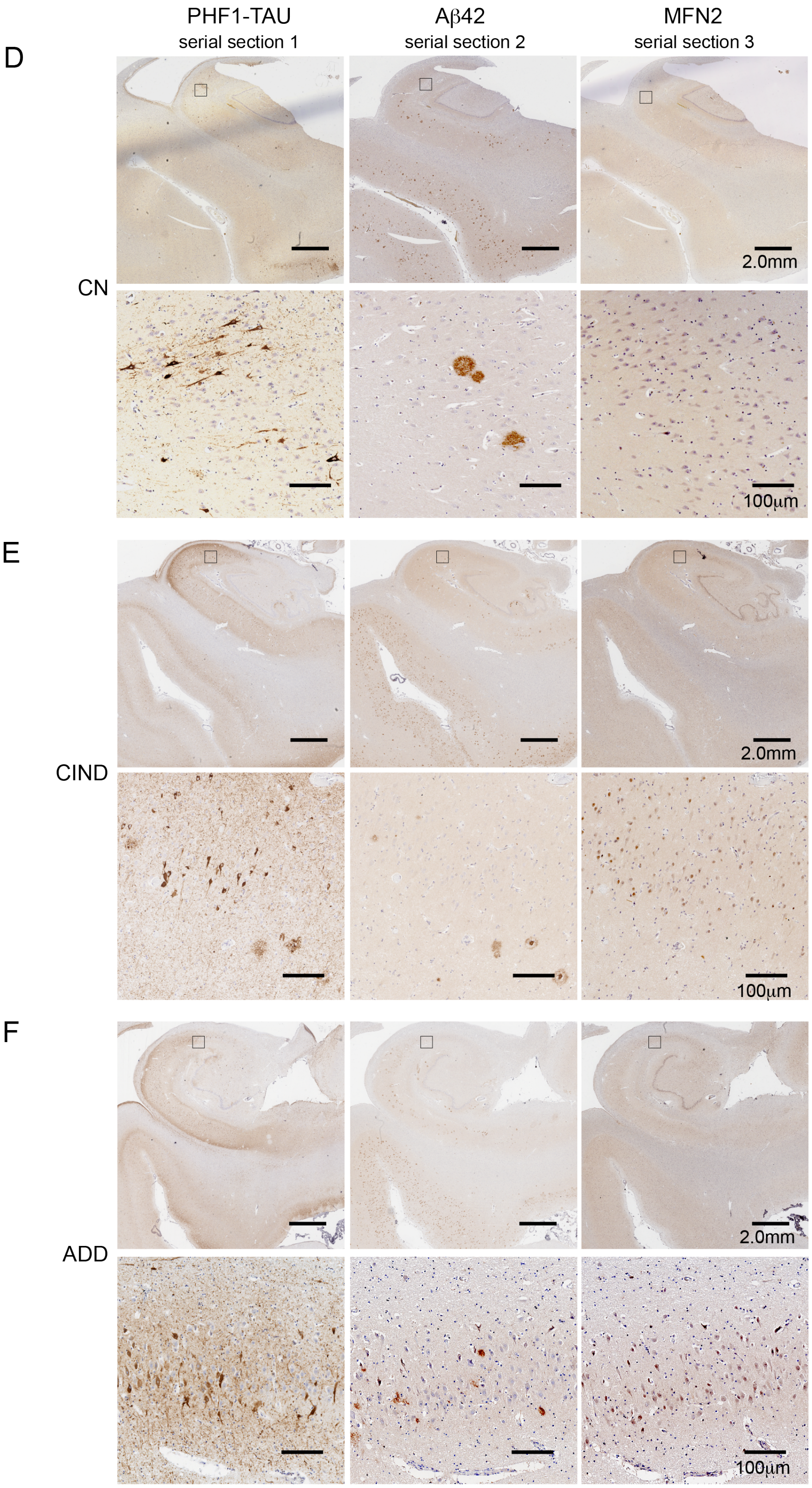

PHF1-TAU
serial section 1
Aβ42
serial section 2
MFN2
serial section 3
D
CN
2.0mm
100μm
E
CIND
2.0mm
100μm
F
ADD
2.0mm
100μm

Figure S6. Neuronal sub-clustering and MFN2 specificity to neurons.
(A) Overlay of tissue samples with neurons alone, colored by subcluster assignment. **(B)** ATAC-seq data from human brain tissue showing localized MFN2 open peaks in neuron cell types only. Gray box represents open MFN2 peaks in neurons alone. **(C)** MFN2 and proteopathy channel relationships across all de novo regions. **(D,E,F)** Validation of PHF1Tau, Aβ42, and MFN2 marker distribution by IHC on the **(D)** CN, **(E)** CIND, and **(F)** ADD tissue serial recuts respectively.
